# Supplementary material for: The interactome of intact mitochondria by cross-linking mass spectrometry provides evidence for coexisting respiratory supercomplexes
Source: Mol Cell Proteomics. 2017 Dec 8;17(2):216–32. doi: 10.1074/mcp.RA117.000470 (PMC5795388; doi:10.1074/mcp.RA117.000470)
Supplement: Supplemental Data [file supp_RA117.000470_133922_0_supp_23978_fzffwf.zip › spectra_annotation/mito_spectra_annotation/467-1-15-1-17-1.pdf]

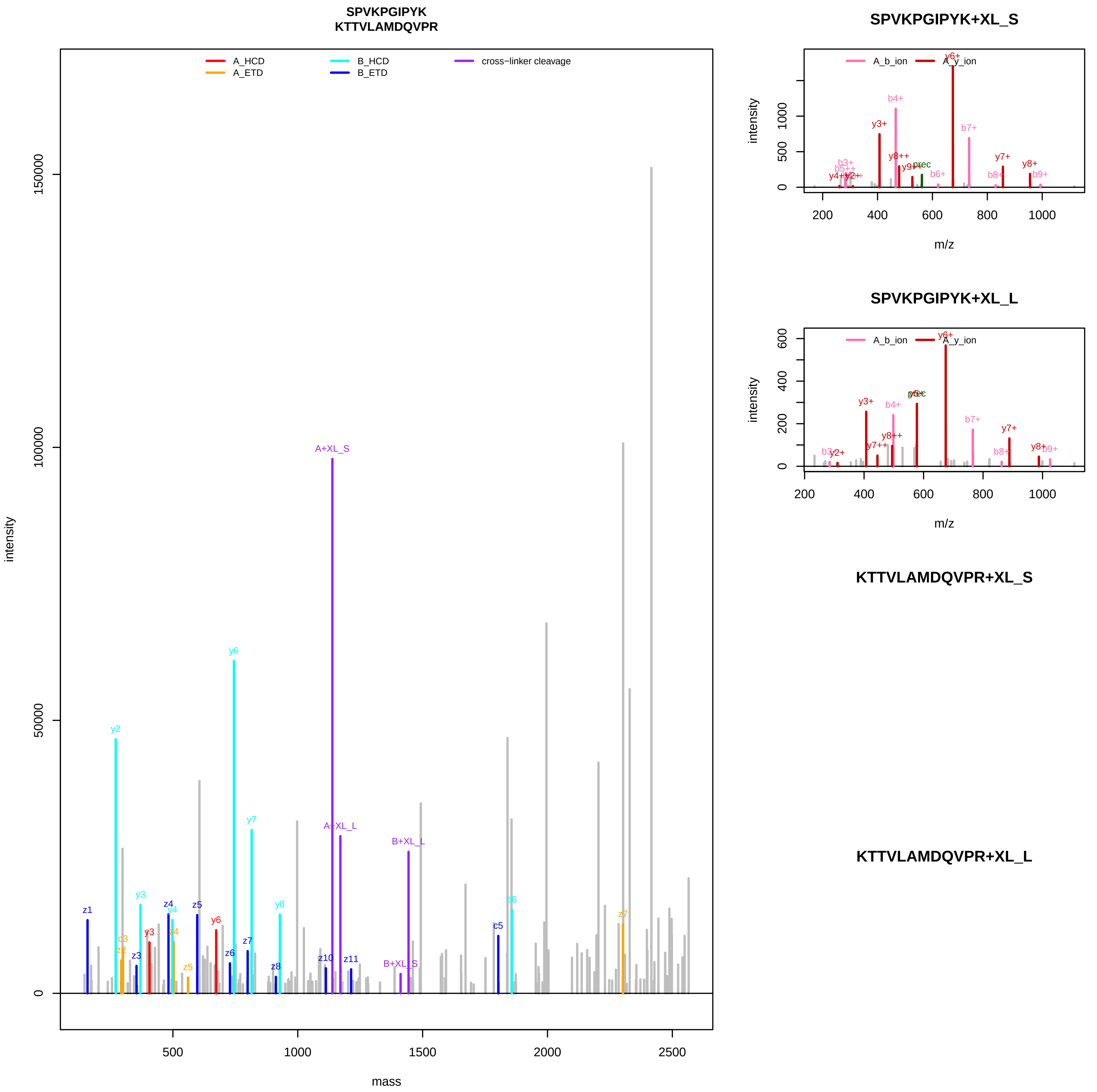
SPVKPGIPYK
KTTVLAMDQVPR
A_HCD
A_ETD
B_HCD
B_ETD
cross−linker cleavage
intensity
mass
0
50000
100000
150000
500
1000
1500
2000
2500
z1
y2
c3
z2
y3
z3
y3
z4
y4
z4
z5
z5
y6
z6
y6
y7
z7
y8
z8
A+XL_S
A+XL_L
z10
z11
B+XL_L
B+XL_S
c5
b6
z7
SPVKPGIPYK+XL_S
A_b_ion
A_y_ion
m/z
SPVKPGIPYK+XL_L
KTTVLAMDQVPR+XL_S
KTTVLAMDQVPR+XL_L
